# HER2 overexpression in urothelial carcinoma with *GATA3* and *PPARG* copy number gains

**DOI:** 10.1093/oncolo/oyae127

**Published:** 2024-06-22

**Authors:** Xiaolin Zhu, Emily Chan, Michelle L Turski, Carlos Espinosa Mendez, Sarah C Hsu, Vipul Kumar, Chase Shipp, Tanya Jindal, Kevin Chang, Courtney Onodera, W Patrick Devine, James P Grenert, Bradley A Stohr, Chien-Kuang Cornelia Ding, Matthew D Stachler, David A Quigley, Felix Y Feng, Carissa E Chu, Sima P Porten, Jonathan Chou, Terence W Friedlander, Vadim S Koshkin

**Affiliations:** Helen Diller Family Comprehensive Cancer Center, University of California San Francisco, San Francisco, CA, United States; Department of Medicine, University of California San Francisco, San Francisco, CA, United States; Helen Diller Family Comprehensive Cancer Center, University of California San Francisco, San Francisco, CA, United States; Department of Pathology, Stanford Medical Center, Stanford, CA, United States; Helen Diller Family Comprehensive Cancer Center, University of California San Francisco, San Francisco, CA, United States; Helen Diller Family Comprehensive Cancer Center, University of California San Francisco, San Francisco, CA, United States; Helen Diller Family Comprehensive Cancer Center, University of California San Francisco, San Francisco, CA, United States; Department of Medicine, University of California San Francisco, San Francisco, CA, United States; Helen Diller Family Comprehensive Cancer Center, University of California San Francisco, San Francisco, CA, United States; Department of Medicine, University of California San Francisco, San Francisco, CA, United States; Helen Diller Family Comprehensive Cancer Center, University of California San Francisco, San Francisco, CA, United States; Department of Medicine, University of California San Francisco, San Francisco, CA, United States; Helen Diller Family Comprehensive Cancer Center, University of California San Francisco, San Francisco, CA, United States; Department of Medicine, University of California San Francisco, San Francisco, CA, United States; School of Medicine, University of California San Francisco, San Francisco, CA, United States; Helen Diller Family Comprehensive Cancer Center, University of California San Francisco, San Francisco, CA, United States; Helen Diller Family Comprehensive Cancer Center, University of California San Francisco, San Francisco, CA, United States; Department of Pathology, University of California San Francisco, San Francisco, CA, United States; Helen Diller Family Comprehensive Cancer Center, University of California San Francisco, San Francisco, CA, United States; Department of Pathology, University of California San Francisco, San Francisco, CA, United States; Helen Diller Family Comprehensive Cancer Center, University of California San Francisco, San Francisco, CA, United States; Department of Pathology, University of California San Francisco, San Francisco, CA, United States; Helen Diller Family Comprehensive Cancer Center, University of California San Francisco, San Francisco, CA, United States; Department of Pathology, University of California San Francisco, San Francisco, CA, United States; Helen Diller Family Comprehensive Cancer Center, University of California San Francisco, San Francisco, CA, United States; Department of Pathology, University of California San Francisco, San Francisco, CA, United States; Helen Diller Family Comprehensive Cancer Center, University of California San Francisco, San Francisco, CA, United States; Department of Urology, University of California San Francisco, San Francisco, CA, United States; Helen Diller Family Comprehensive Cancer Center, University of California San Francisco, San Francisco, CA, United States; Department of Radiation Oncology, University of California San Francisco, San Francisco, CA, United States; Helen Diller Family Comprehensive Cancer Center, University of California San Francisco, San Francisco, CA, United States; Department of Urology, University of California San Francisco, San Francisco, CA, United States; Helen Diller Family Comprehensive Cancer Center, University of California San Francisco, San Francisco, CA, United States; Department of Urology, University of California San Francisco, San Francisco, CA, United States; Helen Diller Family Comprehensive Cancer Center, University of California San Francisco, San Francisco, CA, United States; Department of Medicine, University of California San Francisco, San Francisco, CA, United States; Helen Diller Family Comprehensive Cancer Center, University of California San Francisco, San Francisco, CA, United States; Department of Medicine, University of California San Francisco, San Francisco, CA, United States; Helen Diller Family Comprehensive Cancer Center, University of California San Francisco, San Francisco, CA, United States; Department of Medicine, University of California San Francisco, San Francisco, CA, United States

**Keywords:** HER2, urothelial cancer, *GATA3*, *PPARG*, *ERBB2* amplification

## Abstract

HER2, encoded by the *ERBB2* gene, is an important druggable driver of human cancer gaining increasing importance as a therapeutic target in urothelial carcinoma (UC). The genomic underpinnings of HER2 overexpression in *ERBB2* nonamplified UC are poorly defined. To address this knowledge gap, we investigated 172 UC tumors from patients treated at the University of California San Francisco, using immunohistochemistry and next-generation sequencing. We found that *GATA3* and *PPARG* copy number gains individually predicted HER2 protein expression independently of *ERBB2* amplification. To validate these findings, we interrogated the Memorial Sloan Kettering/The Cancer Genome Atlas (MSK/TCGA) dataset and found that *GATA3* and *PPARG* copy number gains individually predicted *ERBB2* mRNA expression independently of *ERBB2* amplification. Our findings reveal a potential link between the luminal marker HER2 and the key transcription factors GATA3 and PPARG in UC and highlight the utility of examining *GATA3* and *PPARG* copy number states to identify UC tumors that overexpress HER2 in the absence of *ERBB2* amplification. In summary, we found that an increase in copy number of *GATA3* and *PPARG* was independently associated with higher *ERBB2* expression in patient samples of UC. This finding provides a potential explanation for HER2 overexpression in UC tumors without *ERBB2* amplification and a way to identify these tumors for HER2-targeted therapies.

## Teaser

This study found that an increase in copy number of GATA3 and PPARG was independently associated with higher ERBB2 expression in patient samples of urothelial carcinoma, providing a potential explanation for HER2 overexpression in tumors without ERBB2 amplification and a way to identify these tumors for HER2-targeted therapies.

Urothelial carcinoma (UC) is an important cause of cancer morbidity and mortality worldwide. HER2 (encoded by *ERBB2*) is an oncogenic driver frequently altered in multiple cancer types, including breast cancer and malignancies of the gastrointestinal tract,^1^ where HER2-targeted therapies have improved patient outcomes and have received regulatory approvals. *ERBB2* is amplified in approximately 7% of UC tumors with correspondingly high HER2 expression on immunohistochemistry (IHC).^2^ A recent study reported HER2 positivity (IHC 3+ or 2+ with *ERBB2* amplification by fluorescent in situ hybridization) in 15% of UC tumors, while approximately 50% of tumors showed some degree of HER2 expression (IHC ≥ 1+).^2^ Ongoing clinical trials are investigating the efficacy of HER2-targeted antibody-drug conjugates (ADCs) in UC with promising results, using HER2 protein expression as the relevant biomarker.^3,4^ This has led to the recent FDA approval of trastuzumab deruxtecan for patients with unresectable or metastatic HER2-positive (IHC 3+) solid tumors, including UC. However, aside from *ERBB2* amplification,^2^ the genetic basis of HER2 overexpression in UC is not well understood.

**Figure 1.**
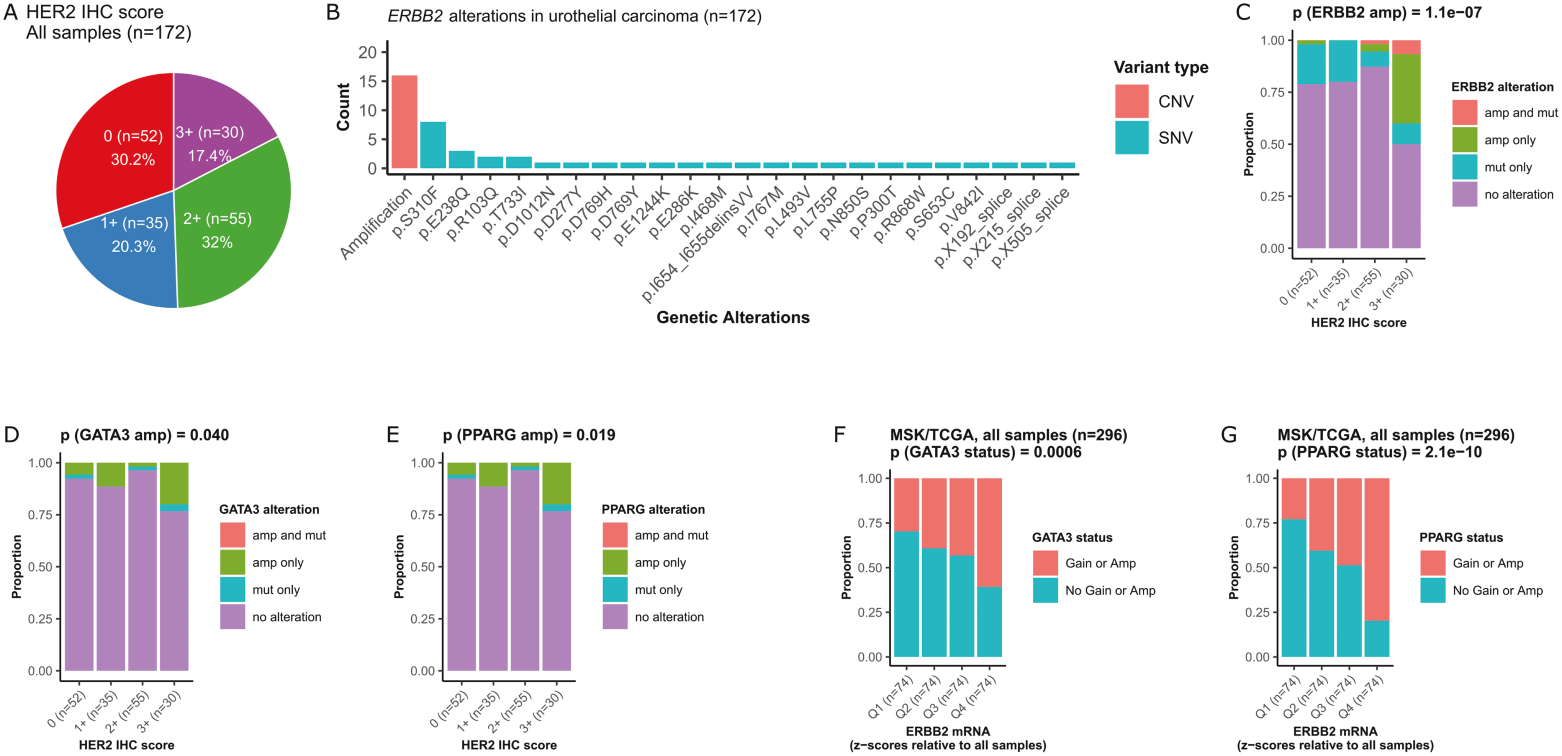
*GATA3* and *PPARG* copy number gains are independently associated with increased *ERBB2* expression in urothelial carcinoma. (A) Distribution of HER2 IHC scores of 172 urothelial carcinoma (UC) samples in the UCSF cohort. (B) *ERBB2* genetic alterations detected on UCSF500, the institutional cancer gene panel. CNV, copy number variant; SNV, single nucleotide variant. (C) *ERBB2* amplifications are enriched in HER2 IHC 3+ UC tumors in the UCSF cohort. (D, E) *GATA3* and *PPARG* amplifications are each enriched in HER2 IHC3+ UC tumors in the UCSF cohort. *P* values were calculated using Fisher’s exact test comparing the proportions of events (ie, *GATA3* and *PPARG* amplifications, respectively) in HER2 IHC 3+ versus 0/1+/2+ samples. (F, G) *GATA3* and *PPARG* gains or amplifications (as defined by the study) are each enriched in muscle-invasive bladder cancer (MIBC) tumors with high *ERBB2* mRNA expression (>4th quartile vs ≤4th quartile) in the MSK/TCGA cohort.

To assess HER2 expression in UC, we retrospectively identified 172 tumor samples of advanced UC (upper tract and muscle-invasive bladder cancer [MIBC]) from 162 patients treated at the University of California San Francisco (UCSF) and sequenced using UCSF500 (an institutional sequencing panel) and evaluated their HER2 expression using IHC (Supplementary Methods). Among the 162 patients, 87 (53.7%) developed metastatic disease during their clinical courses, 32 (19.8%) had upper tract primary tumors, 116 (71.6%) had definitive surgery, and 9 (5.6%) received definitive chemoradiation. The 87 patients with metastatic disease received a median of 2 lines of systemic therapy in the metastatic setting. Using the gastric algorithm^5^ for interpreting HER2 IHC (Ventana, Tuscon, AZ; clone 4B5, ready to use), we found HER2 IHC scores of 0, 1+, 2+, and 3+ in 30.2%, 20.3%, 32%, and 17.4% of samples, respectively (Figure 1A). A higher proportion of IHC 3+ was observed in metastatic versus primary tumors (29.8% vs 11.3%; *P* = .005; Supplementary Figure S1).

To define the genomic profiles of HER2-overexpressing UC, we first examined *ERBB2* alterations (Figure 1B) and found that *ERBB2* amplification was present in 12/30 (40.0%) of HER2 IHC 3+ versus 4/142 (2.8%) of HER2 IHC 0/1+/2+ tumors (Figure 1C; *p* = 1.1 × 10^−7^). Next, to investigate the gene dosage effect for other cancer-related genes (genes included on the UCSF500 panel other than *ERBB2*) on HER2 expression, we evaluated a logistic regression model to predict HER2 IHC 3+ (vs IHC 0/1+/2+) status while adjusting *ERBB2* amplification status as a covariate (Supplementary Methods). We were able to test 500 and 524 cancer-related genes for amplification and deletion, respectively (Supplementary Tables S1 and S2). We identified 46 gene amplifications and 32 gene deletions associated with HER2 IHC 3+ status with a nominal *P* < .05 and searched for their overlaps with the top 200 most frequently amplified and deleted genes, respectively, in the Memorial Sloan Kettering/The Cancer Genome Atlas (MSK/TCGA) MIBC dataset (Supplementary Table S3).^6^ This analysis identified 2 genes, *GATA3* and *PPARG*, both encoding key transcription factors (TFs) in urothelial development and carcinogenesis^7^ and frequently amplified in UC,^8^ that were enriched in HER2 IHC 3+ tumors (Supplementary Table S1; Figure 1D, 1E). Among the 500 genes tested for amplification, *GATA3* was the second (100th percentile) and *PPARG* the 45th (91st percentile) most significant gene independently associated with HER2 IHC 3+ status in the UCSF dataset (Supplementary Table S1). The remaining 44 genes with amplifications and all 32 genes with deletions were not among the top 200 most frequently amplified and deleted genes in the MSK/TCGA dataset, respectively (Supplementary Figure S2A, S2B).

To further examine the *GATA3* and *PPARG* gene dosage effects on *ERBB2* expression, we interrogated the MSK/TCGA MIBC dataset with concurrent DNA and RNA sequencing data accessible through cBioPortal^6,8^ (Supplementary Methods). Because HER2 IHC data were unavailable, we analyzed *ERBB2* mRNA expression as related to *GATA3* and *PPARG* copy number states. We observed higher *ERBB2* mRNA expression in tumors with higher *GATA3* and *PPARG* copy numbers, in all samples (*n* = 296; *P* = .0067 and 1.4 × 10^−10^, respectively; Supplementary Figure S3A, S3B) and in *ERBB2* nonamplified samples (*n* = 280; *P* = .023 and 3.1 × 10^−9^, respectively; Supplementary Figure S3C, S3D). Higher *GATA3* and *PPARG* copy numbers were also each associated with higher mRNA expression of the corresponding genes (*P* = .0021 and <2.2 × 10^−16^, respectively; Supplementary Figure S3E, S3F). *GATA3* and *PPARG* copy number states were each independently associated with *ERBB2* mRNA expression (*P* = .009 and 3.3 × 10^−6^, respectively) in a multivariable linear regression model with *ERBB2* copy number state as a covariate (Supplementary Methods). *ERBB2* mRNA expression was strongly correlated with *GATA3* and *PPARG* mRNA expression, respectively (ρ = 0.55, *P* = 1.4 × 10^−24^ and ρ = 0.55, *P* = 5.2 × 10^−25^, respectively; Supplementary Figure S4A, S4B). Further, we observed enrichment of *GATA3* and *PPARG* copy number gains in MIBC tumors with the top quartile of *ERBB2* mRNA expression, in all samples (*n* = 296, *P* = .0006 and 2.1 × 10^−10^, respectively; Figure 1F, 1G) and *ERBB2* nonamplified samples (*n* = 280, *P* = .0028 and 4.2 × 10^−9^, respectively; Supplementary Figure S5A, S5B).

*GATA3* and *PPARG* encode key TFs driving luminal UC^7^ and are frequently amplified in MIBC (Supplementary Figure S6A, S6B). HER2 is a marker of luminal UC,^7,9^ particularly the luminal unstable (LumU) subtype.^7^ In ReMap,^10^ DNA-binding signals of GATA3 and PPARG (Supplementary Figure S7A, S7B) were observed at the *ERBB2* promoter in multiple cell lines, suggesting potential involvement of GATA3 and PPARG in transcriptional regulation of *ERBB2*, although further studies in UC are needed to test this hypothesis.

**Figure 2.**
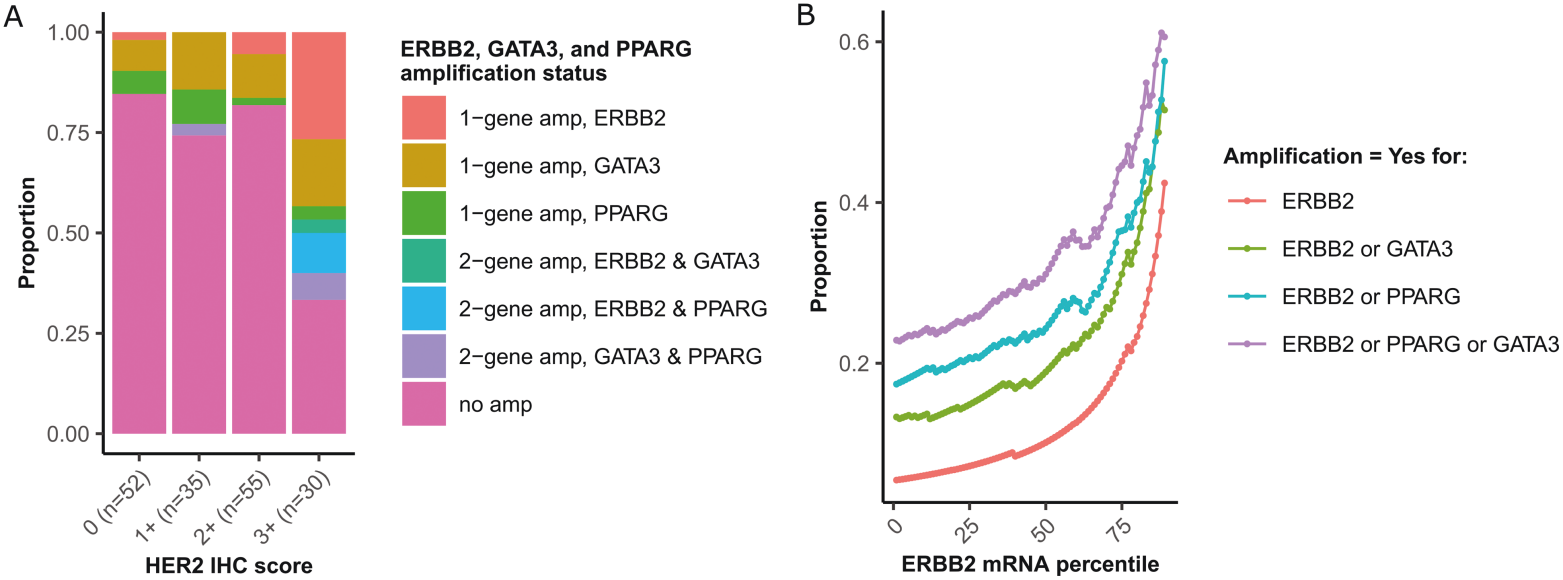
Evaluating *GATA3* and *PPARG* amplifications identifies more tumors with *ERBB2* overexpression. (A) In the UCSF cohort, *GATA3* and *PPARG* amplifications help identify 8 additional HER2 IHC 3+ tumors without *ERBB2* amplification. (B) In the MSK/TCGA cohort, consistently more tumors with *ERBB2* mRNA overexpression are identified by evaluating the amplification status of *GATA3* and *PPARG* in addition to *ERBB2* itself.

To assess the clinical utility of examining gene dosage of *GATA3* and *PPARG* for identifying HER2-overexpressing UC tumors, we first analyzed the UCSF cohort. While 12 of the 30 (40.0%) HER2 IHC 3+ tumors had *ERBB2* amplification (Figure 1C), 8 more HER2 IHC 3+ tumors (8/30; 26.7%) without *ERBB2* amplification were identified by examining the copy number of *GATA3* and *PPARG*, including 5 with *GATA3* amplification alone, one with *PPARG* amplification alone, and 2 with both *GATA3* and *PPARG* amplifications (Figure 2A; Supplementary Figure S8). We next evaluated the MSK/TCGA cohort, where *ERBB2* mRNA levels were used as a surrogate for HER2 expression due to a lack of HER2 IHC data. We found that assessing gene dosage of *GATA3* and *PPARG* in addition to *ERBB2* consistently identified more tumors overexpressing *ERBB2* mRNA (Figure 2B). For example, among MIBC tumors in the top quartile of *ERBB2* mRNA expression, 20.3% displayed *ERBB2* amplification, but analyzing for *GATA3* or *PPARG* amplification identified an additional 24.3% of tumors overexpressing *ERBB2* mRNA. Thus 44.6% of all MIBC tumors in the top quartile of *ERBB2* mRNA expression harbored amplification involving at least one of *ERBB2*, *GATA3*, and *PPARG*. This relationship was consistent across all levels of *ERBB2* mRNA expression (Figure 2B).

In summary, we reported HER2 overexpression in UC tumors with *GATA3* and *PPARG* copy number gains and demonstrated the clinical utility of examining these 2 genes for identifying additional UC tumors overexpressing HER2, particularly when *ERBB2* amplification is not detected. In addition to providing a potential explanation for HER2 overexpression in non-*ERBB2* amplified UC tumors, our findings emphasize the need to look beyond *ERBB2* amplification when using a tumor DNA-based assay to help assess HER2 expression status in UC, an important biomarker for novel HER2-targeted ADCs.

## Supplementary Material

oyae127_suppl_Supplementary_Material

## Data Availability

The data underlying this article will be shared on reasonable request to the corresponding author.
