# Supplementary material for: HER2 overexpression in urothelial carcinoma with GATA3 and PPARG copy number gains
Source: Oncologist. 2024 Jun 22;29(8):e1094–7. doi: 10.1093/oncolo/oyae127 (PMC11299940; doi:10.1093/oncolo/oyae127)
Supplement: oyae127_suppl_Supplementary_Material [file oyae127_suppl_supplementary_material.zip › Supplementary data/TheOncologist.HER2.SuppelmentaryData.revision.docx]

**Supplementary Fig. 1:** HER2 IHC 3+ tumors are more frequently observed in metastatic (**B**) versus primary (**A**) urothelial carcinoma.

**Supplementary Fig. 2:** Volcano plots of the association test results for gene amplifications (**A**) and gene deletions (**B**) using a logistic regression model to predict HER2 IHC status (3+ vs. 0/1+/2+) with gene-level copy number status after accounting for the effect of *ERBB2* amplification status. Outlier genes (those with beta >10 or <-10) were removed before plotting. X-axis: beta (regression coefficient) of the gene tested with *ERBB2* amplification status (yes vs. no) as a covariate. Y-axis: -log10(p) of beta. The green horizontal line indicates the p-value threshold for statistical significance (i.e., -log10(0.05)), and the green vertical lines indicate the effect size threshold for beta (i.e, >1.2 or <-1.2) to help visualize significant genes with large effect sizes.

**Supplementary Fig. 3:** *GATA3* and *PPARG* copy number gains are each associated with increased *ERBB2* mRNA expression in the MSK/TCGA cohort, in all samples (**A** and **B**) and samples without *ERBB2* amplification (**C** and **D**). **E** and **F**, *GATA3* and *PPARG* copy numbers strongly predict their mRNA levels in the MSK/TCGA cohort.

**Supplementary Fig. 4:** *GATA3* (**A**) and *PPARG* (**B**) mRNA levels are each strongly correlated with the *ERBB2* mRNA level in the MSK/TCGA cohort.

**Supplementary Fig. 5:** *GATA3* (**A**) and *PPARG* (**B**) gains or amplifications (as defined by the study) are each enriched in muscle-invasive bladder cancer (MIBC) tumors with high *ERBB2* mRNA expression (>4th quartile versus ≤4th quartile) in samples without *ERBB2* amplifications in the MSK/TCGA cohort.

**Supplementary Fig. 6:** *GATA3* (**A**) and *PPARG* (**B**) are frequently amplified in bladder urothelial carcinoma in the TCGA Pan-Cancer Atlas.

**Supplementary Fig. 7:** In ReMap, a database of DNA-binding experiments, ChIP-seq peaks of GATA3 (**A**) and PPARG (**B**) are observed at the *ERBB2* promoter in multiple cancer cell lines. ChIP-seq, chromatin immunoprecipitation followed by sequencing.

**Supplementary Fig. 8:** *GATA3* amplifications are enriched in HER2 IHC 3+ UC tumors without *ERBB2* amplification in the UCSF cohort (**A**). *PPARG* amplifications are numerically more frequent in *ERBB2* non-amplified HER2 IHC 3+ UC tumors in the UCSF cohort (**B**).

**Supplementary Table 1:** Association testing results for gene-level amplifications. Estimate: logistic regression coefficient of the gene tested with *ERBB2* amplification status included as a covariate to predict HER2 IHC 3+ status. p: p value evaluated with the Wald test.

**Supplementary Table 2:** Association testing results for gene-level copy number losses. Estimate: logistic regression coefficient of the gene tested with *ERBB2* amplification status included as a covariate to predict HER2 IHC 3+ status. p: p value evaluated with the Wald test.

**Supplementary Table 3:** Gene-level copy number states and their frequencies in the MSK/TCGA cohort.

**Supplementary Methods**

**Patient population**

Patients with histologically confirmed urothelial carcinoma (UC, of the upper tract or muscle-invasive bladder cancer [MIBC]) treated at the University of California San Francisco (UCSF) from 2016 through 2022 were included in this analysis if concurrent UCSF500 testing results were available. UCSF500 is a Clinical Laboratory Improvement Amendments (CLIA)-certified next generation sequencing assay, and testing was initiated by the treating physician when clinically indicated (common clinical settings include initial diagnosis of cancer and at disease recurrence or progression). The primary objective of this study was to define the genomic context of HER2-overexpressing UC for all eligible patients; therefore, further patient ascertainment was agnostic to clinical characteristics and treatment history. Approval for this retrospective study was obtained from the UCSF institutional review board (IRB). Baseline clinicopathologic and tumor genomic profiling data for each patient were abstracted from the electronic medical record (EMR) in compliance with the IRB guidelines.

**HER2 immunohistochemistry**

HER2 immunochemistry (Ventana, Tuscon, AZ; clone 4B5, ready to use) was performed using the Ventana Ultra platform (antigen retrieval CC1 36’ at 95oC, incubation time 28 minutes) on the same formalin fixed paraffin embedded (FFPE) tissue block that was used for molecular testing. Interpretation of HER2 IHC was scored by a genitourinary pathologist using established criteria[1] for gastric specimens that are also used for clinical trials of HER2-targeted agents.

**MSK/TCGA data**

We downloaded the MSK/TCGA dataset (n = 476) via cBioPortal[2,3] (<https://cbioportal-datahub.s3.amazonaws.com/blca_msk_tcga_2020.tar.gz>) and conducted genomic and transcriptomic analyses on the 296 samples with both copy number profile and gene expression profile available. Copy number states were defined as per cBioPortal[2,3] (<https://docs.cbioportal.org/user-guide/faq/#what-do-amplification-gain-deep-deletion-shallow-deletion-and--2--1-0-1-and-2-mean-in-the-copy-number-data>). mRNA level was quantified in z-scores relative to all samples (pre-calculated in the dataset downloaded).

**Statistical analysis**

All statistical tests were performed using R 4.2.0 and plots generated using the R package “ggplot2” (<https://ggplot2.tidyverse.org/>). We used Kruskal–Wallis test to compare gene expression (mRNA) levels over >2 groups, and then Wilcoxon test for pairwise comparisons between two groups. We used Fisher’s exact test to compare proportions. Logistic and linear regression models were used to adjust covariates (e.g., *ERBB2* amplification status), for categorical (e.g., HER2 IHC 3+ versus 0/1+/2+ and *ERBB2* mRNA >4^th^ quartile versus ≤4^th^ quartile) and continuous (e.g., *ERBB2* mRNA in z-scores) response variables, respectively.

To determine *ERBB2* amplification status in the UCSF cohort, we used a cutoff gene-level copy number of 6 (amplified if >6, non-amplified if ≤6), extrapolating from the HER2 positivity criteria by in situ hybridization (ISH) in breast cancer[4]. Using this cutoff, we identified 16 of the 172 tumors (9.3%) with amplified *ERBB2*, consistent with the observation in the TCGA Pan-Cancer Atlas study[5].

To identify non-*ERBB2* gene amplifications associated with HER2 IHC 3+ (versus HER2 IHC 0/1+/2+) in the UCSF cohort (n=172), we evaluated the amplification status for each gene except *ERBB2*. For a given gene, the copy number cutoff to call an amplification was set to the median of numerical copy number of that gene in samples with “Amplification” called in the MSK/TCGA dataset (n=300 with both qualitative copy number state calls [i.e., “Deep Deletion”, “Shallow Deletion”, “Diploid”, “Gain”, and “Amplification”] and numerical copy number calls). For example, the copy number cutoff to call an amplification was 3 for *GATA3* and 4 for *PPARG*. We then evaluated each gene individually in a logistic regression model that included *ERBB2* amplification status as a covariate.

[1] Hofmann M, Stoss O, Shi D, Büttner R, van de Vijver M, Kim W, et al. Assessment of a HER2 scoring system for gastric cancer: results from a validation study. Histopathology 2008;52:797–805.

[2] Gao J, Aksoy BA, Dogrusoz U, Dresdner G, Gross B, Sumer SO, et al. Integrative analysis of complex cancer genomics and clinical profiles using the cBioPortal. Sci Signal 2013;6:l1.

[3] Cerami E, Gao J, Dogrusoz U, Gross BE, Sumer SO, Aksoy BA, et al. The cBio cancer genomics portal: an open platform for exploring multidimensional cancer genomics data. Cancer Discov 2012;2:401–4.

[4] Wolff AC, Hammond MEH, Allison KH, Harvey BE, Mangu PB, Bartlett JMS, et al. Human Epidermal Growth Factor Receptor 2 Testing in Breast Cancer: American Society of Clinical Oncology/College of American Pathologists Clinical Practice Guideline Focused Update. J Clin Oncol 2018;36:2105–22.

[5] Cancer Genome Atlas Research Network, Weinstein JN, Collisson EA, Mills GB, Shaw KRM, Ozenberger BA, et al. The Cancer Genome Atlas Pan-Cancer analysis project. Nat Genet 2013;45:1113–20.
