# Supplementary material for: HER2 overexpression in urothelial carcinoma with GATA3 and PPARG copy number gains
Source: Oncologist. 2024 Jun 22;29(8):e1094–7. doi: 10.1093/oncolo/oyae127 (PMC11299940; doi:10.1093/oncolo/oyae127)
Supplement: oyae127_suppl_Supplementary_Material [file oyae127_suppl_supplementary_material.zip › Supplementary data/TheOncologist.HER2.SupplementalFigures.pdf]

Suppl.Fig.1 2
Suppl.Fig.2 3
Suppl.Fig.3 4
Suppl.Fig.4 5
Suppl.Fig.5 6
Suppl.Fig.6 7
Suppl.Fig.7 8
Suppl.Fig.8 9

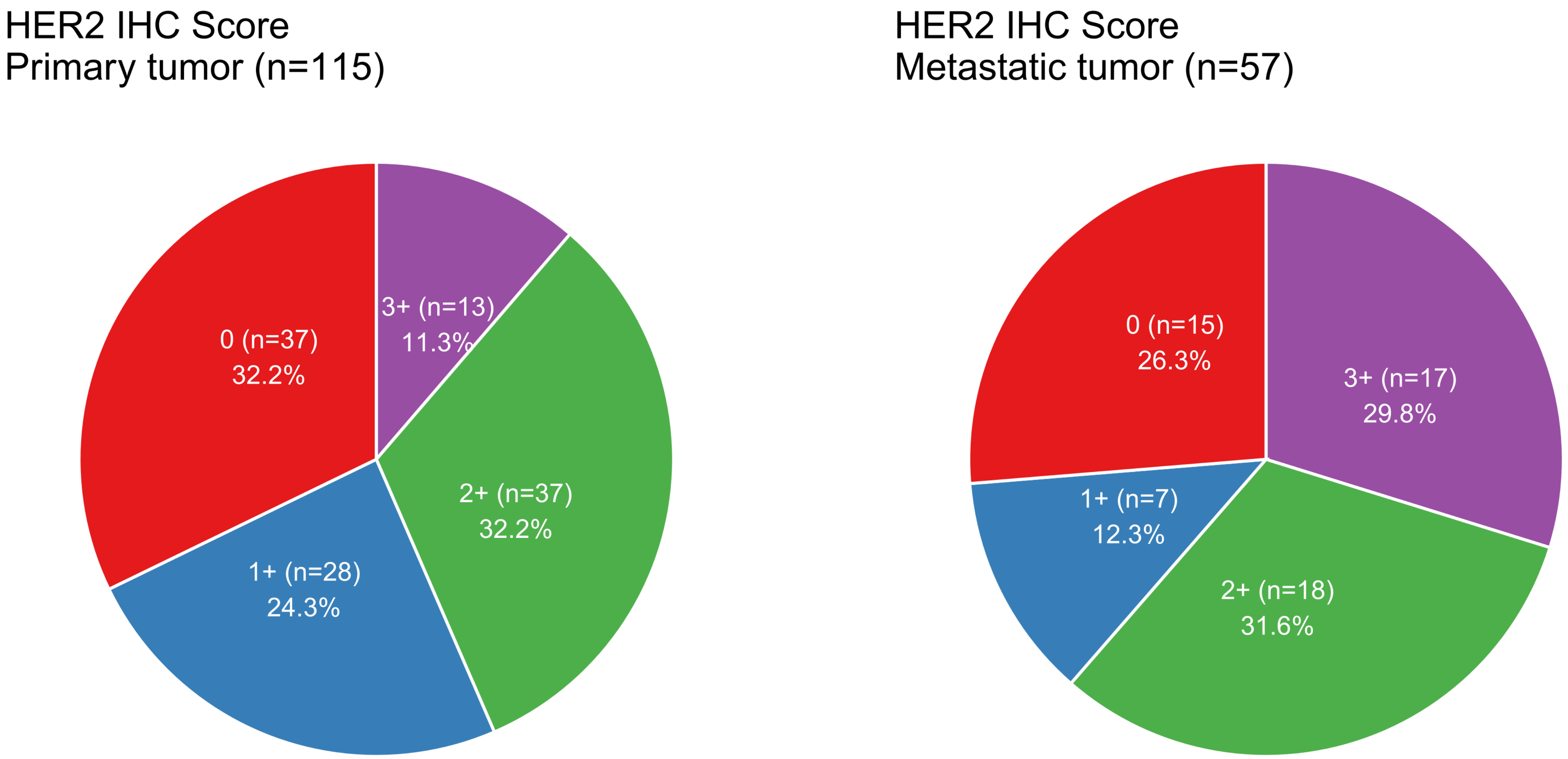

Supplementary Fig. 1

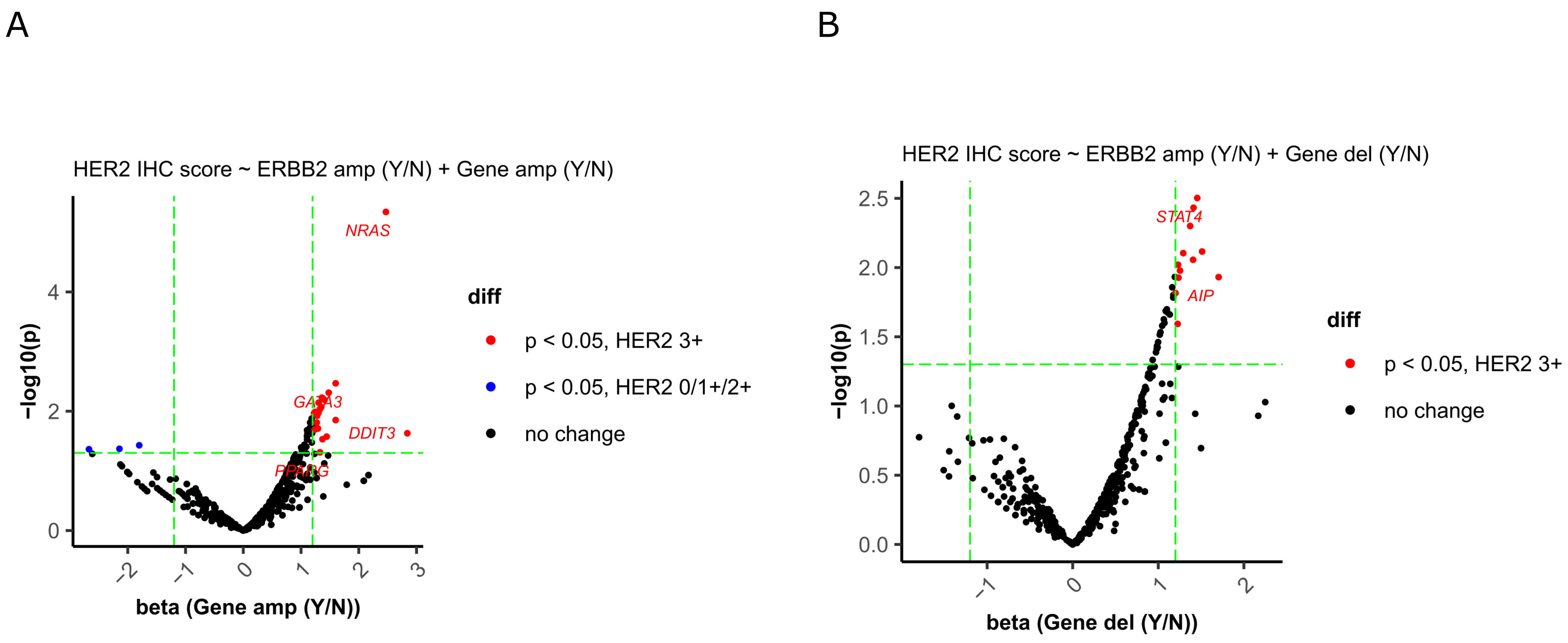

Supplementary Fig. 2

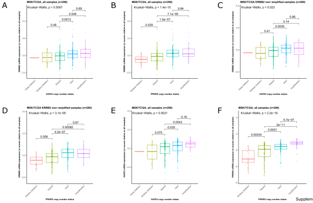

Supplementary Fig. 3

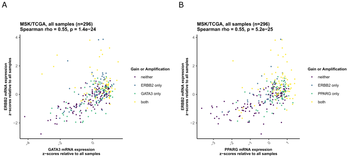

Supplementary Fig. 4

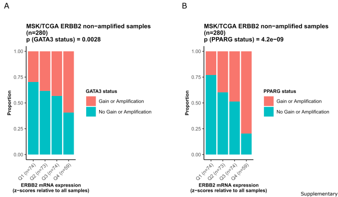

Supplementary Fig. 5

A

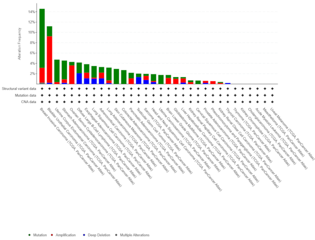

B

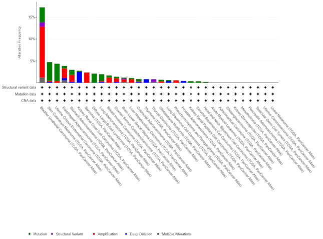

Supplementary Fig. 6

A

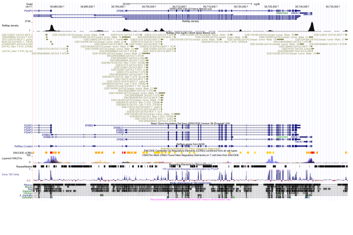

B

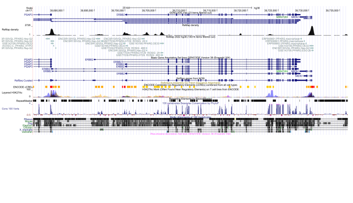

Supplementary Fig. 7

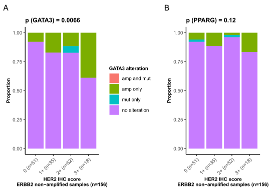

Supplementary Fig. 8
